# Nerve Capping with Porcine Extracellular Matrix Constrains Disorganized Axonal Regrowth: Preliminary Evidence from a Human Nerve Injury Model

**DOI:** 10.3390/cells15211924

**Published:** 2026-10-23

**Authors:** Naveen Arunachalam Sakthiyendran, Caroline J. Cushman, Evan J. Hernandez, Anceslo Idicula, Ian L. Valerio, Brendan J. MacKay

**Affiliations:** 1 Department of Neurosurgery, Boston University Chobanian and Avedisian School of Medicine, Boston, MA 02118, USA; naveenar@bu.edu; 2 School of Medicine, Texas Tech University Health Sciences Center, Lubbock, TX 79430, USA; caroline.cushman@ttuhsc.edu; 3 Department of Orthopaedic Surgery, Texas Tech University Health Sciences Center, Lubbock, TX 79430, USA; evan.j.hernandez@ttuhsc.edu (E.J.H.); anceslo.idicula@ttuhsc.edu (A.I.); 4 Department of Health Sciences, College of Health Sciences, Rush University, Chicago, IL 60612, USA; 5 Department of Hand and Microvascular Surgery, University Medical Center, Lubbock, TX 79415, USA; 6 Division of Plastic and Reconstructive Surgery, Department of Surgery, Massachusetts General Hospital, Harvard Medical School, Boston, MA 02115, USA; ivalerio@mgh.harvard.edu

**Keywords:** nerve cap, neuroma, axonal regeneration, extracellular matrix, histopathology, reconstruction

## Abstract

Peripheral nerve injuries that lack a distal target are prone to symptomatic neuroma formation, yet durable, low-complexity solutions remain limited. We report the first clinical evaluation of a porcine submucosa large-diameter nerve cap (PSLDNC) with paired human histology, contextualized by a previously published preclinical rat study. A 49-year-old man with a mangled hand underwent partial hand amputation with median nerve stump capping using a 7-mm PSLDNC. Eight months later, during revisional trans-radial amputation, the original cap was explanted for histopathology and the median, ulnar, and superficial radial nerves were recapped. Clinically, both residual limb pain and phantom limb pain fell from 39/100 pre-revision to 0 by six and three months, respectively, and remained absent through 15 months; the patient discontinued gabapentin and demonstrated excellent prosthetic function (BAM-ULA 10/10). Histology of the explant showed organized micro-fascicular architecture with minimal collagen deposition and only mild axonal swirling; NF immunostaining demonstrated directional alignment and higher neurofilament density within the capped segment (2326 vs. 945 µm^2^ per 10,000 µm^2^ in a patient control). In that referenced study, capped nerves exhibited fully organized architecture by 12 weeks (axonal swirling score = 0), significantly greater NF-positive area than uncapped controls, minimal local tissue reactivity, and lower pain-related behavioral scores. Together, the human clinical course, human explant histology, and animal histology are consistent with a mechanism in which PSLDNCs confine and guide regenerating axons, allow space for exhaustion of axonal outgrowth, and limit aberrant outgrowth and fibrosis, alongside meaningful relief of neuroma-related pain. Because this is a single patient whose revision also included neuroma resection, nerve re-capping, and intramuscular burial of the stumps, the contribution of the cap cannot be isolated; the absence of an uncapped histologic control and of a clinical comparator further tempers inference. Nonetheless, these data support nerve capping as a feasible, technically straightforward adjunct for neuroma prevention and symptom control, meriting prospective, controlled evaluation and longer-term follow-up.

## 1. Introduction

While treatment paradigms in peripheral nerve surgery continue to evolve, nerve injuries that lack a distal innervation target continue to pose significant clinical challenges. As Wallerian degeneration quickly ensues at the distal end of the injured nerve, the surrounding tissue initiates an inflammatory cascade, which can trigger the formation of neuromas [1]. Neuromas can lead to severe pain, paresthesia, numbness, cold intolerance, and electrical sensitivity, becoming a source of functional and psychological disability for the patient [2,3,4,5]. Moreover, despite the variety of therapies that have been explored in an effort to prevent and/or treat neuromas, there is not yet a universally accepted standard of care [3,6].

Historically, surgical approaches to symptomatic neuromas have primarily involved nerve shortening with stump burial into adjacent muscles, bones, or veins [7]. In the 1980s, Dellon and Mackinnon [8] documented outcomes from neuroma excision followed by implantation of the nerve end into muscle [9]. Other authors also detailed techniques involving neuroma excision and implantation into proximal bones [10] or veins [9,11,12]. While some cases have shown modest success, these methods have not universally proven effective in symptom improvement, with simple excision appearing less effective compared to other surgical approaches. The excision and implantation technique, while widely used, is somewhat passive in neuroma treatment: it removes the affected nerve segment but fails to address the nerve stump’s regenerative potential or establish a pathway for neuroma-free regeneration. These traditional methods have yielded inconsistent results, with many patients experiencing recurrent neuroma-related pain, highlighting the inadequacy of passive techniques alone [7,8,10,11,12,13,14,15,16,17,18,19,20,21,22]. Inevitably, the nerve’s distal end will attempt to regenerate in its relocated position, potentially leading to the formation of a recurrent symptomatic neuroma. Studies have reported that 46% of patients who had a neuroma excised saw no improvement in symptoms and only 33–40% of patients were satisfied with treatment after burial into surrounding bone or musculature [1,23,24,25]. These limitations have driven interest in more active strategies to redirect or contain regenerating axons.

Two such approaches—targeted muscle reinnervation (TMR) and regenerative peripheral nerve interface (RPNI)—are increasingly favored in modern microsurgery [26,27]. TMR is a procedure in which the transected nerve is connected to adjacent/nearby functional motor nerves and the associated muscle. This procedure allows the nerve to purposefully re-innervate in a controlled fashion and avoids the development of a neuroma [28,29]. RPNI, on the other hand, is a procedure which involves rerouting a transected nerve to an adjacent/nearby muscle target to allow the nerve to reinnervate and regain functional capacity [30]. These procedures offer advantages, as nerves that were otherwise dysfunctional following an amputation can be functionally regained, allowing for restoration of function by coaptation [26]. However, these techniques present notable challenges, including the complexity of the surgical procedure, the impact of scar tissue and incomplete nerve regeneration on the success of the intervention, and the variability in patient outcomes [26]. Additionally, procedures such as RPNI are still in the experimental stage and long-term outcomes data on durability and efficacy remain limited.

In parallel, nerve capping has emerged as a less invasive, technically straightforward strategy that directly addresses the regenerative behavior of the nerve stump. Capping the proximal stump with a biocompatible barrier promotes contact guidance through scaffolding and limits axonal exposure to neurotrophic and inflammatory cues in surrounding tissue, thereby discouraging disorganized regrowth [7]. This barrier effect has also been observed in photo-sealed nerve caps: Scott et al. used autologous vein or crosslinked amniotic membrane sealed over transected rat sciatic nerves, demonstrating histologically organized axonal architecture and the absence of neuroma formation [31].

Several materials have been investigated in animal models for use as nerve caps, demonstrating promising outcomes. As an alternative, biocompatible devices, such as nerve caps, are designed to isolate the nerve stump from inflammatory stimuli, and have demonstrated efficacy in preclinical models by reducing aberrant axonal growth and associated neuropathic pain [21]. Despite encouraging animal-model findings, which use small-diameter nerve caps, clinical evidence evaluating nerve capping, particularly using large-diameter nerve caps such as the Axoguard Porcine Submucosa Large-Diameter Nerve Cap (PSLDNC), remains significantly limited.

In this proof-of-concept case study, we present the first reported clinical evaluation of a PSLDNC in a patient treated with nerve cap implantation. Explantation of the PSLDNC occurred incidentally during a later revisional trans-radial amputation and this allowed direct histologic assessment of the capped nerve. This study seeks to clarify the potential role of PSLDNC in improving neuroma management and neuropathic pain relief through histology analysis and clinical outcomes, offering peripheral nerve surgeons a novel strategy in neuroma management.

## 2. Case Presentation

A 49-year-old male with no significant prior medical history sustained a severe crush injury to his left upper extremity during a workplace accident, resulting in extensive soft tissue damage, multiple open fractures, and a severely mangled hand. During the initial emergent procedure, the first three metacarpals, hanging only by a skin bridge, were amputated, and thorough debridement was performed to minimize infection risk. Despite multiple subsequent irrigation and debridement procedures, the fourth digit remained dysvascular, dysfunctional, and nonviable, prompting an intraoperative decision for partial hand amputation at the fourth metacarpal level. Given the transection of the median nerve at the amputation site, a 7 mm Axoguard Porcine Submucosa Large-Diameter Nerve Cap (PSLDNC; Axoguard Nerve Cap^®^, Axogen Corporation, Alachua, FL, USA) was placed over the proximal nerve stump. The device is a porcine small-intestine submucosa (SIS) extracellular matrix cap configured as a tubular implant with one open end, one sealed (capped) end, and internal channels, together with a suturable end tab; the transected nerve was drawn into the lumen and secured within the cap.

**Figure 1:**
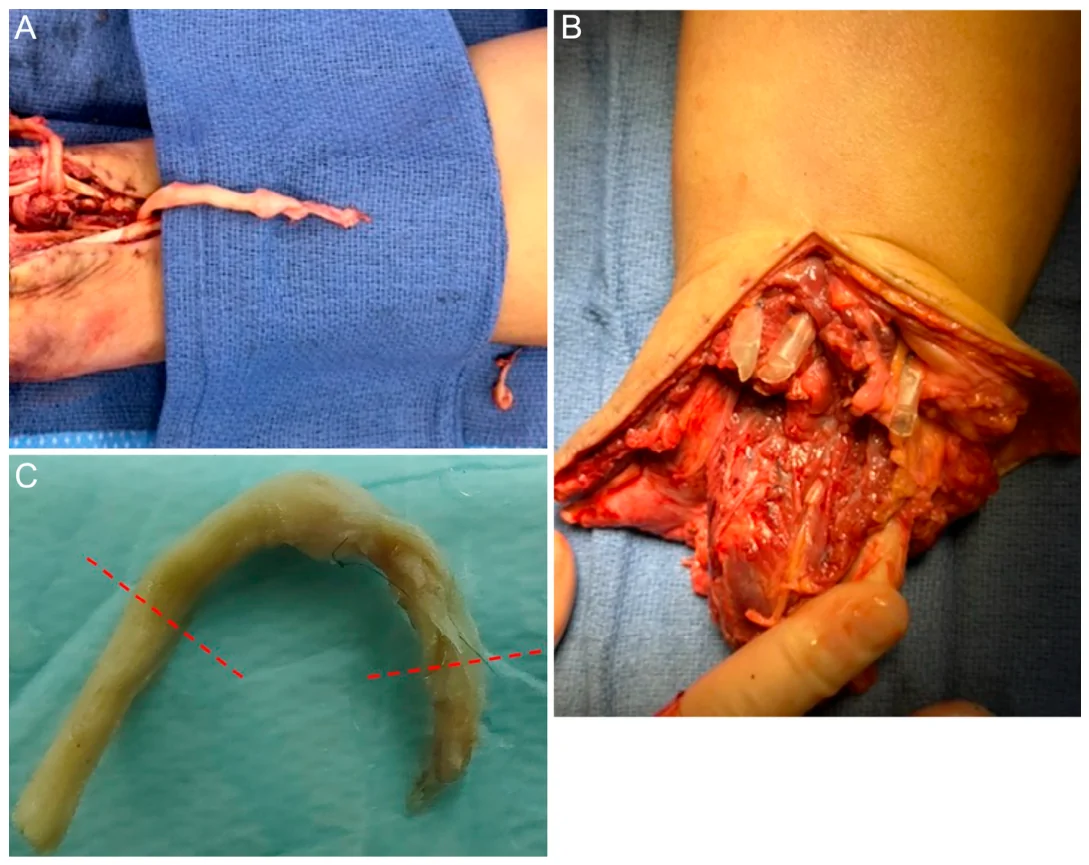
Intraoperative and Anatomical Images. (**A**) Removal and exposing of median nerve cap. (**B**) Subsequent addition of median, ulnar, and superficial branch of radial nerve caps. (**C**) Isolated left median nerve with red dashed lines indicating the boundaries of the nerve cap for parafin embedding.

Although compliant with postoperative instructions and rehabilitation, the patient experienced persistent stiffness and decreased range of motion over the subsequent eight months. Due to ongoing functional limitations and discomfort, at 246 days post-operative the patient ultimately elected to undergo a revisional transradial amputation proximal to the wrist. During this subsequent procedure, the previously capped median nerve stump was surgically exposed, the PSLDNC was explanted, and the specimen was sent for histological analysis (Figure 1). Following explantation, new PSLDNCs were applied to the median nerve (7 mm), ulnar nerve (7 mm), and superficial branch of the radial nerve (SBRN) (5 mm), with all three capped nerves carefully buried within adjacent musculature to facilitate isolation. Of note, this patient did not present with, and was not clinically diagnosed with, a discrete symptomatic end-neuroma prior to revision: no focal tender mass, positive Tinel sign over a palpable neuroma, or imaging-confirmed neuroma was documented. The initial nerve cap was therefore placed prophylactically at the time of amputation to prevent symptomatic neuroma formation, and the subsequent revision was driven by functional limitation and stiffness together with residual limb pain (39/100) rather than by a formally diagnosed neuroma. Explantation of the prophylactic cap at revision provided the opportunity for the histologic evaluation reported here.

## 3. Methods

During the preparation of this manuscript, written informed consent for participation and for the publication of clinical details and images was obtained from the patient [32].

### 3.1. Case Timeline

The chronological course of care is summarized below (all intervals are relative to the index injury and initial amputation):

Day 0—Workplace crush injury to the left upper extremity with extensive soft-tissue damage, multiple open fractures, and a mangled hand; emergent amputation of the first three metacarpals (retained only by a skin bridge) with thorough debridement.

Early postoperative period—Multiple staged irrigation and debridement procedures; the fourth digit remained dysvascular and nonviable, prompting partial hand amputation at the fourth-metacarpal level. The median nerve, transected at the amputation site, was capped prophylactically with a 7-mm porcine submucosa large-diameter nerve cap (PSLDNC) over the proximal stump.

Months 1–8—Persistent stiffness and decreased range of motion with residual limb discomfort; the patient remained compliant with rehabilitation.

Day 246 (~8 months)—Elective revisional transradial amputation proximal to the wrist. The original median-nerve PSLDNC was explanted and sent for histopathology; new PSLDNCs were applied to the median (7 mm), ulnar (7 mm), and superficial radial (5 mm) nerves and buried within adjacent musculature.

Three months post-revision—Phantom limb pain decreased from 39/100 to 0.

Six months post-revision—Residual limb pain decreased from 39/100 to 0.

Twelve and 15 months post-revision—Patient remained free of both residual and phantom limb pain; gabapentin (300 mg daily) was discontinued; functional outcome was excellent (BAM-ULA 10/10).

### 3.2. Human Median Nerve Sample Preparation

The explanted median nerve cap specimen was immediately placed in 10% neutral buffered formalin and processed in entirety to paraffin on a three-hour per station cycle using a Sakura Tissue-Tek VIP6 processor. Sections were cut at 4 μm on a Leica RM2255 semi-automated microtome with Katana low-profile blades and mounted onto adhesive microscope slides. Hematoxylin and Eosin (H&E) and Masson’s Trichrome (MT) stains were applied using standard protocols.

### 3.3. Histologic Quantification

Histopathological evaluation was performed by a licensed pathologist using H&E, MT, and Neurofilament (NF) staining. Specifically, three levels from the nerve specimen were evaluated for axonal swirling, scored using 0 to 4 scoring criteria, where 0 was defined as no axonal swirling and 4 was defined as marked axonal swirling.

### 3.4. Immunohistochemical Staining Evaluation

Immunohistochemistry was performed on 4-μm paraffin sections for two axonal markers: Neurofilament (clone 2F11; mouse monoclonal; Dako M0762; dilution 1:8000; working concentration 0.017 μg/mL) and Neurofilament 200 (rabbit polyclonal; Abcam ab8135; dilution 1:6000). Pretreatment included endogenous-peroxidase inhibition with 3% hydrogen peroxide, proteinase K digestion, and heat-induced epitope retrieval as appropriate for each antibody. Antigen–antibody complexes were detected with the Dako EnVision+ horseradish-peroxidase system, visualized with 3,3′-diaminobenzidine (DAB+) chromogen, and counterstained with hematoxylin. Negative controls were processed in parallel with omission of the primary antibody. All stained slides were digitized at 20× magnification on an Aperio ScanScope AT2(Leica Biosystems, Vista, CA) whole-slide scanner for qualitative and quantitative analysis.

Neurofilament-positive axonal area was quantified from the digitized slides using ImageJ/Fiji (version 2.3.0/1.53f51; Java 1.8.0_172, 64-bit; National Institutes of Health, Bethesda, MD, USA). Five non-overlapping 100 × 100 μm regions of interest (ROIs) were sampled at random within the capped nerve specimen, and an equal number of equivalently sized ROIs were sampled within the normal proximal nerve stump that served as the intra-specimen reference. For each ROI, color deconvolution was used to isolate the DAB (brown) chromogen, and a manual intensity threshold was applied to segment NF-positive signal from background; the identical threshold and ROI dimensions were applied uniformly to specimen and reference ROIs to limit measurement bias. NF expression was expressed as the DAB optical density of the segmented NF-positive signal normalized to a 10,000 μm^2^ field. Qualitative histologic assessment and the semiquantitative axonal-swirling score (0–4 scale; 0 = no swirling, 4 = marked swirling) across three levels of the specimen were performed by a single board-certified pathologist. Owing to the macroscopic differences between the explanted capped specimen and the native-nerve reference, formal blinding of the pathologist to specimen origin was not feasible; to mitigate observer bias, the quantitative NF analysis relied on fixed, operator-independent thresholding parameters applied identically to all ROIs.

## 4. Results

### Patient Outcomes and Histological Evaluation

Postoperatively, the patient reported significant pain improvement, self-rating his pain as 4/10 during initial follow-up visits, without numbness or paresthesia. Residual limb pain progressively decreased from a preoperative score of 39 (on a 0–100 scale) to 0 by the six-month follow-up, with sustained relief through 12- and 15-month evaluations. Phantom limb pain followed a similar course, declining from 39 preoperatively to 0 by three months, with no recurrence reported at later visits. At the 15-month follow-up, the patient remained free of both residual and phantom limb pain and had successfully discontinued previously prescribed gabapentin (300 mg daily). Functional outcomes, assessed using the Brief Activity Measure for Upper Limb Amputees (BAM-ULA), demonstrated excellent prosthetic use, with the patient scoring 10/10.

**Figure 2:**
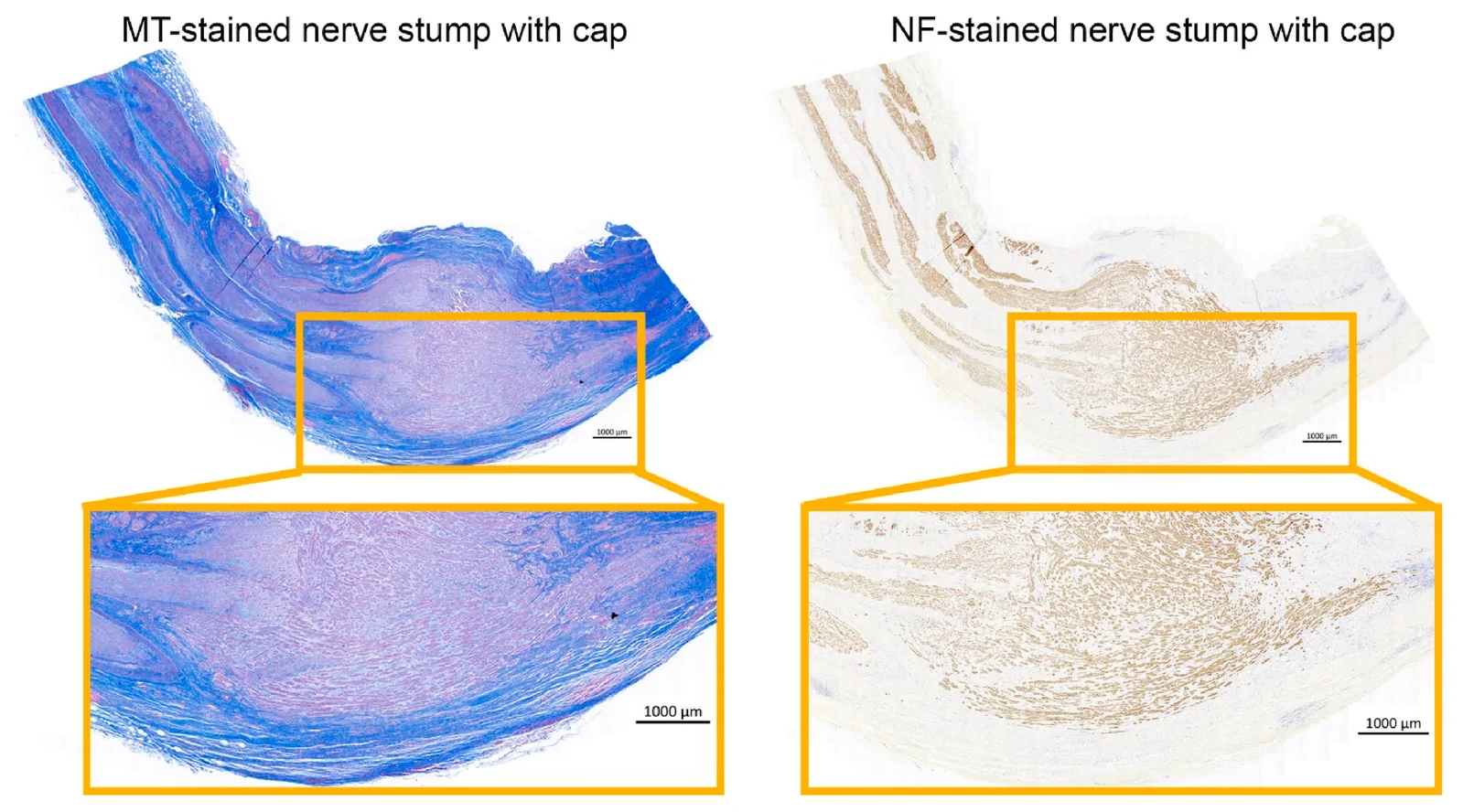
Patient median nerve histology. Masson’s Trichrome (MT) (shown on the left side)-stained sections showed similar collagen deposition (blue) dispersed between axon bundles (pink), indicating expected histoarchitecture of a remodeling nerve. This histoarchitecture suggests a bulbous neuroma had not formed on the nerve stump. Neurofilament (NF) stain (shown on the right side) showed directional alignment of axons (brown) with mild axonal plane deviation within the nerve cap. There were organized fascicles traveling from the proximal to distal end of the nerve which is indicative of limited and controlled neuroma growth.

**Figure 3:**
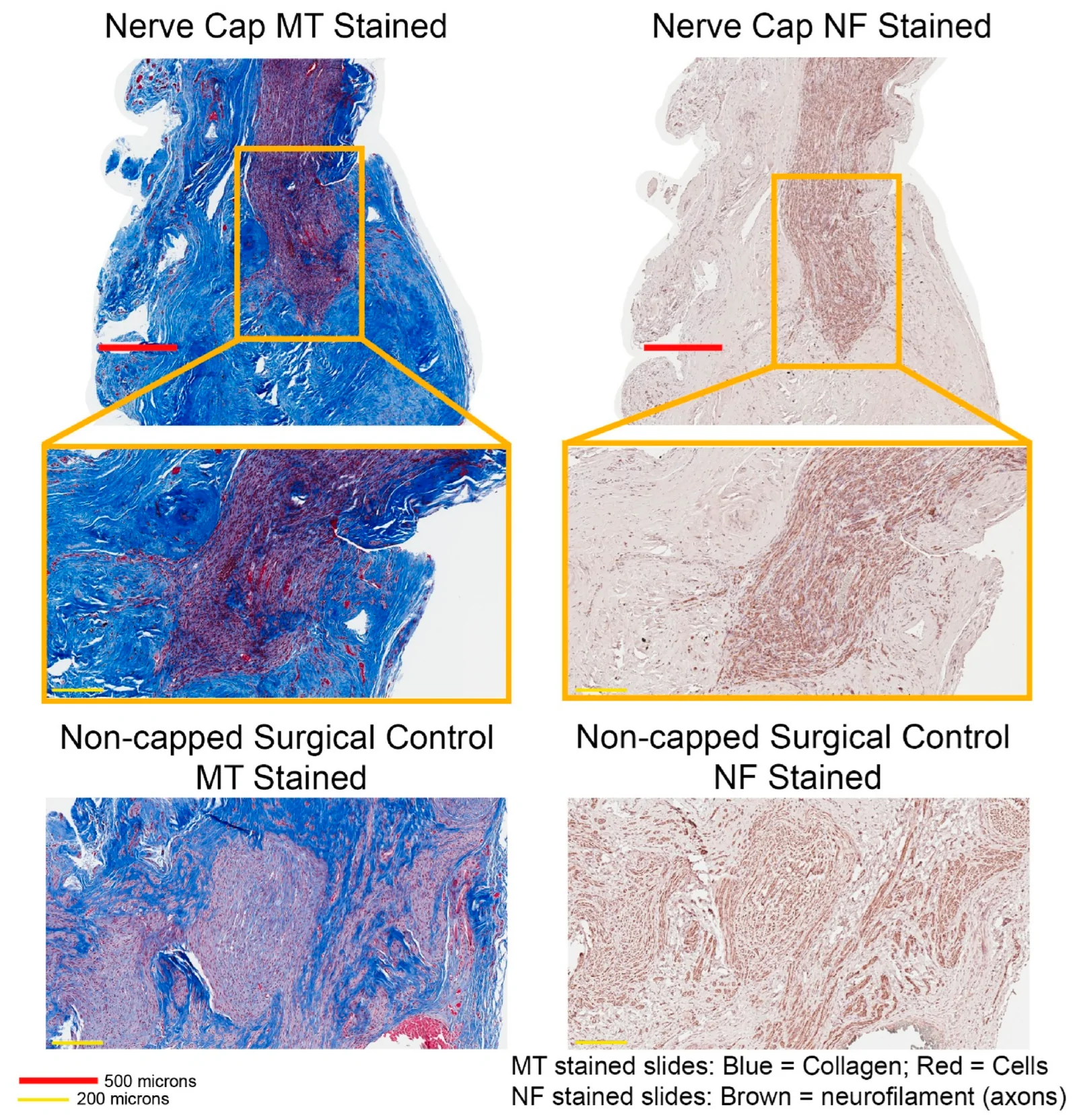
Rat in-vivo model comparison of nerve cap-treated and Non-Capped Nerve Stumps. Representative longitudinal sections of capped and non-capped nerve stumps demonstrating collagen deposition and axonal organization. Masson’s Trichrome (MT) staining (shown on the left side) of a nerve cap-treated stump shows organized axonal bundles (red) interspersed within collagen matrix (blue), with preserved longitudinal architecture. Neurofilament (NF) staining (shown on the right side) of a nerve cap-treated specimen demonstrates directional axonal alignment (brown) within the cap. Insets highlight maintained fascicular organization. Bottom panels depict non-capped surgical control specimens. MT staining reveals disorganized collagen deposition consistent with neuroma formation, while NF staining demonstrates irregular axonal growth and loss of parallel alignment. Red scale bars = 500 μm; yellow scale bars = 200 μm. MT: blue = collagen, red = cellular elements; NF: brown = neurofilament-positive axons. Permission was obtained from *Tork, S., Faleris, J., Engemann, A., Deister, C., DeVinney, E., & Valerio, I. L. (2020). Application of a Porcine Small Intestine Submucosa Nerve Cap for Prevention of Neuromas and Associated Pain. Tissue engineering. Part A, 26(9–10), 503–511. https://doi.org/10.1089/ten.TEA.2019.0273 for appropriate use of this figure* [21].

Histopathological examination of the explanted nerve cap specimen revealed predominantly organized nerve fibers arranged into bundles or micro-fascicular patterns. Masson’s trichrome staining demonstrated minimal collagen deposition or fibrosis surrounding the capped nerve segment (Figure 2). The pathologist evaluation of NF-stained slides identified axonal alignment with only minimal deviation, graded as scores ranging from 1 (minimal swirling) to 2 (mild swirling). The overall axonal swirling score was assigned a score of 2 (mild swirling). Quantitative image analysis, based on five randomly selected fields, revealed a neurofilament density of 2326 µm^2^ per 10,000 µm^2^ within the capped specimen, compared with 945 µm^2^ per 10,000 µm^2^ in a healthy nerve control from the same patient. Because the comparator was the patient’s own healthy proximal nerve rather than an uncapped amputation neuroma, the density difference alone cannot establish neuroma suppression; rather, the organized, directionally aligned, micro-fascicular architecture is consistent with contained axonal regeneration and paralleled the patient’s lack of neuropathic pain. These clinical findings were consistent with the histological results seen in a previously published animal model using small-diameter nerve caps of the same design and material (Figure 3) [21].

## 5. Discussion

Neuromas are a debilitating consequence of peripheral nerve injury and remain a leading cause of post-amputation pain and disability, which limits prosthetic use in patients [9,22]. Given the significant impact on quality of life, a wide range of nonoperative and operative interventions have been explored to mitigate neuroma formation and symptoms. While nonoperative treatments such as neuromodulation [33] and serial chemical injection [34,35] have been employed, they often require repeated treatments and have variable efficacy. As a result, surgical management remains the mainstay treatment, with nerve capping becoming an area of interest for surgical management of painful neuromas.

In a rat model, Yan et al. used polyglycolic acid conduits as caps, showing that capping altered the regenerative state of transected nerves and suppressed pain-related markers like substance P and c-fos, suggesting a molecular basis for neuroma prevention [36]. Similarly, Pan et al. investigated the use of long acellular nerve allografts (ANAs) with no distal target as caps in a rat neuroma model. They found that the ANA caps effectively exhausted axonal regeneration and modulated gene expression in dorsal root ganglia, including decreased expression of regeneration- and pain-associated genes such as Bdnf, cfos, and Gal [37]. This suggests that ANAs with no distal target can serve as effective nerve caps to prevent neuroma formation and associated pain. Additionally, a study by Acevedo Cintrón et al. demonstrated that limited nerve regeneration across long ANAs coincides with changes in blood vessel morphology and the development of a pro-inflammatory microenvironment [38].

Recently, decellularized extracellular matrix-based caps, particularly porcine small-intestine submucosa, have gained attention for their remodeling potential. The Axoguard PSLDNC, used in our patient case, represents a new class of nerve caps designed for larger-diameter peripheral nerves (2–7 mm). The PSLDNC is thought to provide a barrier between the regenerating axons and the external soft-tissue environment and sufficient length for the nerve to grow in a more aligned fashion, thus minimizing pathological interactions among damaged axons.

Histological evaluation of the explanted nerve cap in this case supports its proposed mechanism of action. The specimen showed organized micro-fascicular axonal bundles, minimal collagen deposition, and directional alignment of axons on H&E and NF staining, consistent with controlled, non-pathologic regeneration. Our findings were mirrored in a rodent study by Tork et al., where the tibial nerves of 57 Sprague Dawley rats were transected and transposed to the lateral hind leg [21]. In small-diameter (1.5 mm) capped rat tibial nerves, axonal swirling scores reached 0 in all animals by 12 weeks, indicating fully organized axonal architecture. Uncapped rat tibial nerves of the surgical control group showed minimal to mild axonal swirling in 71.5% of explants (Figure 3) [21]. Although some mild swirling was seen in the capped human sample, the difference between the animal model having no swirling and the human model showing mild swirling is likely due to the regenerative burden of the larger-diameter nerve that was capped in the human specimen. The neurofilament density of our clinical specimen was substantially higher in the capped nerve (2326 μm^2^ per 10,000 μm^2^) compared to a healthy control nerve from the same patient (945 μm^2^). Because this control is a normal nerve rather than an uncapped amputation neuroma, the density difference cannot by itself demonstrate inhibition of neuroma formation and may instead reflect the biology of an actively regenerating nerve; the organized, aligned, micro-fascicular pattern is the more informative finding. The PSLDNC used here is composed of the same porcine SIS extracellular matrix as the device evaluated by Tork et al., differing principally in diameter, and its internal-channel (“bifurcation”) geometry corresponds most closely to the chambered configuration tested in that study. In the chambered arm, Tork et al. reported a mean NF optical density of approximately 7858 µm^2^ per 10,000 µm^2^ (vs. 6289 µm^2^ for the spiral cap and ~3062 µm^2^ for uncapped controls); because cap geometry influenced axonal outgrowth in that model, the chambered value is the most appropriate point of reference for the device implanted here [21]. Additional histological evaluation reported in this rat study indicated that host tissue response remained minimal, with inflammation and fibroplasia scores consistently in the non-irritant range (<3.0), and histology confirmed limited collagen deposition and favorable cap integration [21]. This in vivo animal histology analysis strengthens the hypothesis that PSLDNCs inhibit neuroma formation by guiding axons through a confined architecture, minimizing aberrant regrowth and fibrotic encapsulation.

Pain after amputation of a limb is a common symptom and is separated into two types of pain including residual limb pain (RLP) and phantom limb pain (PLP). PLP is clinically defined as the perception of pain or discomfort in a limb that no longer exists. On VAS, PLP decreased from 39 pre-revision to 0 by three months (Table 1). RLP or “stump pain”, on the other hand, is pain that originates from the actual site of the amputated limb. It is most common in the early post-amputation period and tends to resolve with wound healing. Unlike PLP, RLP is often a manifestation of an underlying source, such as nerve entrapment, neuroma formation, surgical trauma, ischemia, skin breakdown, or infection. RLP decreased from 39 pre-revision to 0 by six months following the revision procedure, which included neuroma resection, removal of the original PSLDNC, re-capping of the transected median, ulnar, and superficial radial nerve ends, and intramuscular burial of all three stumps (Table 1). Thus, the overall revision with re-capping was associated with substantial PLP and RLP relief. A similar behavioral pain response reported by Tork et al. showed that in the rat tibial transposition model, rats in the groups with a nerve cap showed significantly less pain response than rats without a nerve cap, indicating the nerve caps’ ability to reduce or eliminate symptomatic neuroma formation [21].

Taken together, the quantitative density measurements and traditional staining results provide one of the first objective, human-derived correlates between histological architecture and clinical outcome after nerve capping and explantation, reinforcing the role of nerve caps as active scaffolds for neuroma-free axonal regeneration. These results mirrored those seen in the rat tibial transposition model by Tork et al. [21]. Other recent studies have further confirmed this concept with capping [1,39]. Specifically, Faust et al. demonstrated that nerve capping improved overall axonal organization but limited axonal regrowth within the grafts without fibrous overgrowth or hyper-cellularity [39]. In 2024, a systematic review identified only 146 human cases treated with various types of nerve caps, most involving smaller-caliber sensory nerves and non-biologic materials [22]. This report represents the first clinical case detailing implantation of a PSLDNC in a human subject with a major peripheral nerve injury.

In examining the capped nerve directly, there was organized micro-fascicular architecture with minimal axonal swirling and limited collagen deposition, findings concordant with the patient’s clinical improvement and consistent with the cap’s proposed role in preventing neuroma formation. Several limitations constrain the inferences that can be drawn. First, this is a single patient, so treatment effect cannot be separated from natural history, regression of early postoperative pain, or placebo response, and no statistical inference is possible. Second, the revision procedure bundled several interventions known to reduce neuroma pain—neuroma resection, removal and replacement of the cap, and intramuscular burial of all three stumps—so the independent contribution of the PSLDNC to the observed pain relief cannot be isolated. Third, the histologic comparator was the patient’s own healthy proximal nerve rather than an uncapped amputated stump; the neurofilament-density difference may therefore reflect normal regenerative biology rather than neuroma suppression, and the qualitative architecture is the more interpretable signal. The favorable clinical and histologic findings are encouraging but hypothesis-generating only. Prospective, controlled studies with uncapped (and ideally TMR/RPNI) comparator arms, blinded histologic assessment, and longer follow-up are needed to delineate the independent contribution of nerve capping.

### Tork et al. Animal Model

The animal data shown in Figure 3 are not original to the present report; they are reproduced, with permission, from the previously published preclinical study of Tork et al. [21], and are included here to provide mechanistic context for the human histologic findings. The experimental design of that study is summarized below as reported by the original authors. No new animal experiments were performed for this case report.

In that study, a tibial neuroma-transposition (TNT) model in Sprague Dawley rats was used, adapted from the method of Dorsi et al. [40]. The tibial nerve was transected and the proximal stump transposed to the lateral hindlimb, where it was either left untreated (surgical control) or capped with one of two porcine small-intestine submucosa (pSIS) configurations—a chambered nerve cap (CNC) or a spiral nerve cap (SNC); an open-tube configuration was also evaluated. Animals were allocated across treatment groups with a target of eight animals per group at each time point and were assessed at two postoperative time points, 8 and 12 weeks; a subset of animals was euthanized early owing to device erosion through the skin, reducing the final per-group sample sizes. The pSIS material corresponds to the same class of porcine submucosa used in the large-diameter nerve cap reported in the present human case.

Neuropathic pain behavior was assessed weekly by applying a standardized series of ten punctate stimuli to the treated and contralateral (untreated) hindlimbs; each trial was scored 0–2 (0 = no response, 1 = slow paw withdrawal, 2 = brisk withdrawal, shaking, licking, or vocalization) and summed across the ten trials per session. Histologic analysis sampled three levels approximately 200 μm apart, with serial sections stained by hematoxylin and eosin, Masson’s trichrome, and neurofilament (NF) immunohistochemistry. Axonal swirling was graded by a veterinary pathologist on the same semiquantitative 0–4 scale used for the human specimen, and NF optical density was quantified to index axonal content. Group comparisons used nonparametric methods—Kruskal–Wallis testing with post hoc Tukey and Kramer–Nemenyi multiple-comparison tests, with Fisher’s exact test for the frequency of axonal swirling and Pearson correlations between swirling, optical density, and behavior; data were reported as mean ± standard error of the mean at a 0.05 significance threshold.

In the reported results, the capped groups showed more organized neural architecture and reduced aberrant regeneration relative to untreated controls. By 12 weeks, the untreated surgical-control group exhibited significantly more frequent axonal swirling (approximately 71.5% of animals) than the capped groups, whereas the CNC and SNC groups showed no identifiable axonal swirling in 100% of animals (Fisher’s exact test, *p* = 0.02), alongside a greater NF-positive signal and reduced pain-related behavior in capped versus uncapped stumps. These preclinical observations—organized microfascicular regrowth, greater NF-positive area, and reduced pain behavior with capping—parallel the histologic and clinical pattern observed in the explanted human PSLDNC specimen, supporting the biological plausibility of the present findings while remaining distinct, species-separate evidence.

## 6. Conclusions

Taken together, the oriented fibers, absence of severe axonal swirling, and reduction in pain support the intended function of PSLDNCs of guided, controlled axonal outgrowth and limitation of neuroma formation, which is consistent with how a small-diameter nerve cap functioned in a previous animal study. Future studies should evaluate the long-term clinical and histologic outcomes of nerve capping and the role of caps in preserving the nerve end for potential reinnervation procedures (e.g., TMR), rather than routine explantation.

## Figures and Tables

**Table 1:** Residual and Phantom Limb Pain of Patient at Various Follow-Ups.

	Pre-Operative	2 Week Post-Op	1 Month Post-Op	3 Month Post-Op	6 Month Post-Op	9 Month Post-Op	12 Month Post-Op	15 Month Post-Op
Residual Limb Pain	39	28	25	28	0	0	0	0
Phantom Limb Pain	39	5	5	0	0	0	0	0

## Data Availability

Data can be found in the manuscript text, tables and figures.
